# Prediction of the Setting Properties of Calcium Phosphate Bone Cement

**DOI:** 10.1155/2012/809235

**Published:** 2012-07-10

**Authors:** Seyed Mahmud Rabiee, Hamid Baseri

**Affiliations:** Department of Mechanical Engineering, Babol Noshirvani University of Technology, Mazandaran, Babol 47148-71167, Iran

## Abstract

Setting properties of bone substitutes are improved using an injectable system. The injectable bone graft substitutes can be molded to the shape of the bone cavity and set in situ when injected. Such system is useful for surgical operation. The powder part of the injectable bone cement is included of **β**-tricalcium phosphate, calcium carbonate, and dicalcium phosphate and the liquid part contains poly ethylene glycol solution with different concentrations. In this way, prediction of the mechanical properties, setting times, and injectability helps to optimize the calcium phosphate bone cement properties. The objective of this study is development of three different adaptive neurofuzzy inference systems (ANFISs) for estimation of compression strength, setting time, and injectability using the data generated based on experimental observations. The input parameters of models are polyethylene glycol percent and liquid/powder ratio. Comparison of the predicted values and measured data indicates that the ANFIS model has an acceptable performance to the estimation of calcium phosphate bone cement properties.

## 1. Introduction

Bioactive calcium phosphates such as hydroxyapatite (HA) Ca_10_(PO_4_)_6_(OH)_2_, tricalcium phosphate (TCP) Ca_3_(PO_4_)_2_, tetracalcium phosphate (Ca_4_P_2_O_9_), and dicalcum phosphate (DCP) CaHPO_4_ have been widely applied for hard tissue substitute materials, due to their good biocompatibility and bioactivity [1–4]. Many studies have evidenced the excellent biocompatibility of calcium phosphates (CaPs) and their favorable interaction with hard tissue [5, 6]. The shapes of CaPs for the practical uses are classified into the dense and porous CaP blocks [7, 8], the powders and granules [9], the CaP coating [10, 11], and the CaP cement [12, 13]. The hardened forms of CaPs have a major disadvantage. One of the shortcomings is the difficulty of fitting into the defects. The particulate form of CaP can easily fill the defects; however, it migrates or disperses into surrounding tissue [14, 15]. One of the major improvements in CaPs in recent years is the development of an injectable system. The injectable bone graft substitutes can mold to the shape of the bone cavity and set in situ when injected. Such systems should shorten the surgical operation time, reduce the damaging effects of large muscle retraction, decrease the size of the scars and diminish postoperative pain. It also allows the patient to achieve rapid recovery in a cost-effective manner [15, 16]. Calcium phosphate cements (CaPCs) were the first injectable bone filling developed for bone substitute applications [17]. Brown and Chow prepared the first CPC in 1985 contained TTCP and DCP as the solid phase. After mixing with water, the cement forms HA as the only final product. In an aqueous environment at 37°C, CPC transformed to HA which is more similar to biological apatite than sintered HA formed at high temperatures [18, 19].

Many different CaPC formulas have been studied, but most of them form HA as final product [20]. The CaPCs, now available on the market, are too stable to permit material degradation and bone ingrowth in a limited period of time, at least for the first years [21].

As a matter of fact, the properties of a CPC, such as injectability, setting time, and final strength, can be modulated through variation in powder composition, liquid phase, liquid-to-powder ratio, and ageing conditions [20, 21]. Furthermore, a number of organic and polymeric additives [22–24] have been used with the aim to improve the properties of CaPCs. Poly ethylene glycol (PEG) is being widely used in drug delivery systems because of its hydrophilicity and biocompatibility [25, 26], but there are few published articles on the use of PEG/CaP composites. In this study, the effect of PEG on the CaPC was investigated. The different concentration of PEG was used as the liquid phase, and the powder component consisted of TCP, DCPA and calcium carbonate. The surface morphology of cement powder and phase detection of cements were performed using scanning electron microscopy and X-ray diffraction. Also, the injectability, setting behavior, and compressive strength of this cement were measured.

With the development of computer technology and artificial intelligence methods, the estimation in nonlinear problems has become an effective method. Guild and Bonfield [27] developed a predictive model for hydroxyapatite-reinforced polyethylene composite using a finite-element analysis method. The predictive model can be used to investigate the micromechanical behavior of the material. In another research [28], they developed a finite-element model to improve the ductility at high-volume fractions. Cao et al. [29] developed a modified artificial neural network (ANN) to model the nonlinear relationship between ultrasonic precipitation parameters and the HA content. Input parameters are temperature, reaction time, and ultrasonic power. The improved model for processing dataset and selecting its topology developed using the Levenberg-Marquardt training algorithm and trained with comprehensive dataset of HA nanoparticles collected from experimental data. In the previous paper by the authors [30], a backpropagation neural network was developed to predict the mechanical strength and the setting times in an individual type of HA bone cement. This model had two parameters of the NaH_2_PO_4_·2H_2_O solution as liquid phase and the liquid/powder ratio of the cement. Results show that the model had an acceptable performance to estimate the setting times and mechanical strength in HA bone cement.

In this research, the cements consist of both powder and liquid phases. The powders consist of calcium carbonate, dicalcium phosphate anhydrous and *β*-tricalcium phosphate. These materials are similar to mineral phase of bone [1–4]. Also, polyethylene glycol (PEG) is being widely used in biomedical applications due to its hydrophilicity and biocompatibility [25, 26]. The different concentration of PEG was used as the liquid phase because PEG solution can improve the properties of a CPC, such as injectability, and setting time. Then, an adaptive neurofuzzy inference system is used to correlate the effective input parameters to mechanical properties, setting times, and injectability of synthesized CaPC using the data generated based on experimental observations. Also, unused results of the experiments were compared with those of the ANFIS predictions, and best architectures were designed for minimal error.

## 2. Experimental Procedure 

### 2.1. Preparation of Cements

In this research, the powder phase is a mixture of *β*-TCP, DCPA (Merck) and CaCO_3_, (Merck). *β*-TCP was synthesized by the solid-state reaction method. A mixture of 2 moles of DCPA and 1 mole of CaCO_3_ were heated to 1200°C for 6 hr and then cooling in a box furnace [14]. After choosing the proper amounts of starting materials, the powder cement was prepared by mixing the starting materials together for 1 hr. The liquid phase was an aqueous solution of PEG (MW = 400, Merck). In order to investigate the effects of PEG concentration, various amounts (0, 4, 8, 12, 16, and 20%, w/v) of PEG were dissolved in a distilled water/ethanol (1/1, v/v) composite solution by stirring for 6 hr at room temperature. The effect of liquid/powder ratio (*L*/*P*) was also studied in four ratios of 0.3, 0.35, 0.4, and 0.45 mL/g. The cement samples were prepared by mixing the powders and the liquids together in a mortar for about 1 min. 

Morphological evaluation of the powder cement was performed by SEM utilizing a Stereoscan 360 microscope (Leica, Cambridge, UK). The composition of the cements was analyzed by means of powder X-ray diffraction using a Philips 3710-MPD control equipped with Cu *K*_*α*_  radiation. Data were collected from 2*θ* = 25–45° with a step size of 0.02° and a normalized count time of 1 s/step. 

To obtain the crystallinity of the cements, a minimum of the (002) apatite peaks (2*θ* = 25.9°) of the specimens after setting time were recorded. The angular width of the (002) diffraction peak (*B*) was measured at 1/2 of the height of the maximum intensity above the background. The *B* value was corrected for instrumental broadening by Warren's method using the following expression [31, 32]:

(1) $$\begin{array}{c} B^{2}=b^{2}+\beta^{2}, \end{array}$$

where *b* and *β* are the instrumental broadening and the corrected with of the peak, respectively. Because peak width inversely correlates with crystallite size and lattice perfection (i.e., the smaller the width, the larger and/or less strained the crystal) the 1/*β* value was calculated to relate the XRD data more directly to these crystal parameters.

### 2.2. Measurement of the Cement Properties

Setting times of these cements were measured according to the ISO 9917 standard for dental silicophosphate cement. Setting times of the samples were measured by using a Vicat apparatus. Any sample was considered set when a 400 gram mass loaded to a needle with a tip diameter of 1 mm did not make any visible impression on the surface of the sample. Injectability of the cement was evaluated using a disposable syringe and taking out the paste through the syringe by hand pressure. The syringes had a capacity of 5 mL with an opening nozzle size of 2 mm in diameter. As mixed paste of 4 gram was added into the syringe and extruded after 5 min. The paste was extruded from the syringe by hand until it was unable to inject entirely. The weight of the injected paste was then measured, and injectability was calculated using the following equation [15, 33]:

(2) $$\begin{array}{c} \%\;\text{Injectability}=\frac{\text{Paste}\;\text{weight}\;\text{expelled}\;\text{syringe}}{\text{total}\;\text{paste}\;\text{weight}\;\text{before}\;\text{injecting}}\times 100. \end{array}$$

The mechanical strength of the synthesized cements was evaluated as follows: cement paste was packed in a cylindrical plastic mold with 6 mm in diameter and 12 mm in length. After setting the cements were incubated in 100% relative humidity at 37°C for 7 days. The specimens were crushed with a cross-head speed of 1 mm/min using an Instron Universal Testing Machine (6025). The compressive strength (*C*_*s*_) of the sample was calculated from the following formula:

(3) $$\begin{array}{c} C_{s}=\frac{2F}{\pi dh}, \end{array}$$

where *F* is the peak load (N), *d* is the diameter (mm), and *h* is the thickness (mm) of sample. The maximal compression load at failure was obtained from the recorded load-deflection curves. For each measurement, an average of five samples was taken.

## 3. Adaptive Neurofuzzy Inference System (ANFIS)

An adaptive neurofuzzy inference system gives the mapping relation between the input and output data by using hybrid learning method to determine the optimal distribution of membership functions [34]. Both artificial neural network (ANN) and fuzzy logic (FL) are used in ANFIS architecture. Basically, five layers are used to construct this inference system. Each ANFIS layer consists of several nodes described by the node function. The inputs of present layers are obtained from the nodes in the previous layers.

**Figure 1:**
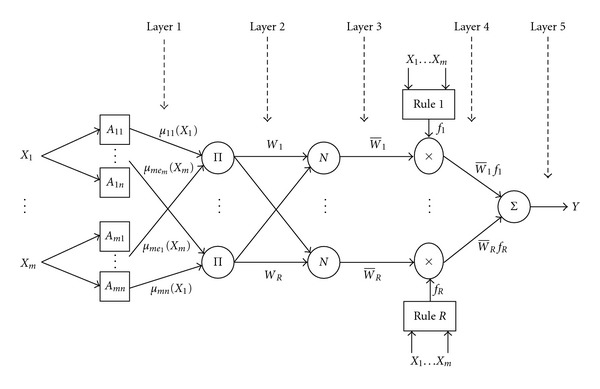
Basic structure of ANFIS.

Figure 1 shows the basic ANFIS structure for a system with *m* inputs (*X*_1_,…, *X*_*m*_), each with *n* membership functions (MFs), a fuzzy rule base of *R* rules, and one output (*Y*). The network consisting of five layers is used for training Sugeno-type fuzzy interface system (FIS) through learning and adaptation. The number of nodes (*N*) in layer 1 is the product of numbers of inputs (*m*) and MFs (*n*) for each input, that is, *N* = *mn*. The number of nodes in layers 2–4 depends on the number of rules (*R*) in the fuzzy rule base. Five Layers of ANFIS model are as follow.

*Layer* 1 (*fuzzification layer*). It transforms the crisp inputs *X*_*i*_ to linguistic labels (*A*_*ij*_, like small, medium, large etc.) with a degree of membership. The output of node *ij* is expressed as follows:

(4) $$\begin{array}{c} O_{ij}^{1}=\mu_{ij}\left(X_{i}\right),\;i=1,\ldots ,m,j=1,\ldots ,n, \end{array}$$

where *μ*_*ij*_ is the *j*th membership function for the input *X*_*i*_. Several types of MFs are used, for example, triangular, trapezoidal, and generalized bell function. In this study it selected a generalized bell function by trial and error, as follows:

(5) $$\begin{array}{c} \mu \left(X\right)=\frac{1}{1+{\left|\left(X-c\right)/a\right|}^{2b}}, \end{array}$$

where *a* and *b* vary the width of the curve, and *c* locates the center of the curve. The parameter *b* should be positive. These parameters are named as premise parameters.

*Layer* 2 (*product layer*). For each node *k* in this layer, the output represents weighting factor (firing strength) of the rule *k*. The output *W*_*k*_ is the product of all its inputs as follows:

(6) Ok2=Wk=μ1e1(X1)μ2e2(X2),…,μmem(Xm), k=1,…,R, e1,e2,…,em=1,…,n.

*Layer* 3 (*normalized layer*). The output of each node *k* in this layer represents the normalized weighting factor ${\overset{-}{W}}_{k}$ of the kth rule as follows:

(7) $$\begin{array}{c} O_{k}^{3}={\overset{-}{W}}_{k}=\frac{W_{k}}{W_{1}+W_{2}+\cdots +W_{R}} \end{array}$$

*Layer* 4 (*defuzzification layer*). Each node of this layer gives a weighted output of the first-order TSK-type fuzzy if-then rule as follows:

(8) $$\begin{array}{c} O_{k}^{4}={\overset{-}{W}}_{k}f_{k}, \end{array}$$

where *f*_*k*_ represents the output of *k*th TSK-type fuzzy rules as follows:

(9) If (X1  is  A1e1) and (X2  is  A2e2) and…and (Xm  is  Amem),then  fk=∑i=1mpieiXi+rk,

where *p*_*ie*_*i*__ and *r*_*k*_ are called consequent parameters and *e*_1_, *e*_2_,…, *e*_*m*_ = 1,…, *n*, *k* = 1,…, *R*.

*Layer* 5 (*output layer*). This single-node layer represents the overall output (*Y*) of the network as the sum of all weighted outputs of the following rules:

(10) $$\begin{array}{c} O^{5}=Y=\sum_{k=1}^{n}{\overset{-}{W}}_{k}f_{k}. \end{array}$$

## 4. Results and Discussion

### 4.1. Characterization

**Figure 2:**
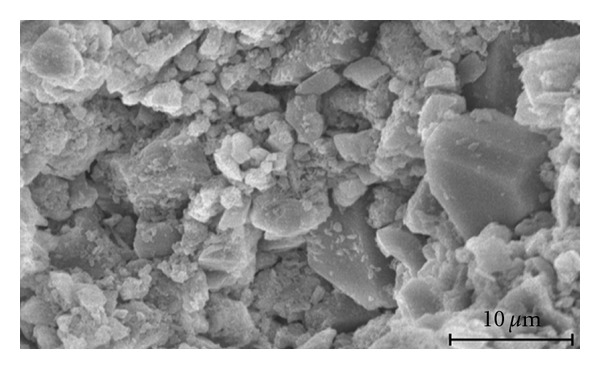
SEM micrograph of the cement powder before mixing with the liquid phase.

The morphology of the cement powder before adding the liquid phase is shown in the SEM image reported in Figure 2. A few big plate-like crystals due to DCPA are clearly distinguishable from the synthesized *β*-TCP and CaCO_3_ particles, which display an average size of 3–7 *μ*m. 

**Figure 3:**
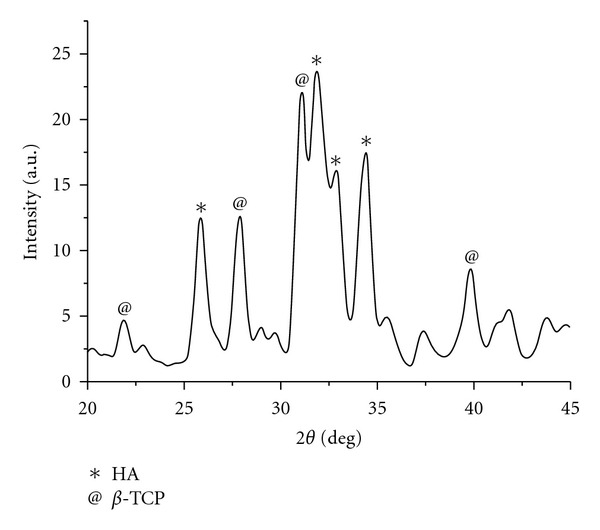
X-ray diffraction pattern of cement with 4% PEG and *L*/*P* = 0.4 mL/g after 7-day incubation at 37°C and 100% relative humidity.

XRD patterns of cements showed that the presence of PEG does not affect appreciably the final phase of the cements that exhibits the diffraction reflections characteristic of *β*-TCP and HA. But, the PEG had an influence on the resulting of crystallinity of the cements. The calculation of the crystallinity index (1/*β*) of the cements for *L*/*P* = 0.4 cc/gr with 0%, 4%, 12%, and 20% run yielded crystallinity of about 2.50, 1.04, 0.78 and 0.45 degree, respectively. When the liquid cement no contain of any percents of PEG, the final phase of cement had a crystalline phase of apatite (1/*β* = 2.78 degree) and when the present of PEG, the final phase of the cements had low crystallinity. With adding PEG, the cements were converted to bone-like poor crystalline apatite. The X-ray pattern of the cement with liquid contains 4% PEG and *L*/*P* = 0.4 cc/gr after 7-day incubation at 37°C and 100% relative humidity which is reported in Figure 3.

### 4.2. Data Preprocessing for ANFIS Development

Before the ANFIS can be trained and the mapping learnt, it is important to process the experimental data into patterns. Training/testing pattern vectors are formed. Each pattern is formed with an input condition vector and the corresponding target vector.

The scale of the input and output data is an important matter to consider, especially when the operating ranges of process parameters are different. The scaling or normalizing ensures that the ANFIS will be trained effectively, without any particular variable skewing the results significantly. As a result, all of the input parameters are equally important in the training of the neural network. The scaling is performed by mapping each term to a value between *new* min and *new* max using the following equation:

(11) $$\begin{array}{c} V_{\text{nor}}=new\min\;+\;\frac{V_{i}-V_{\min}}{V_{\max}-V_{\min}}\left(new\max\;-\;new\min\right), \end{array}$$

where *V*_nor_ is the normalized value, *V*_*i*_ is the value of a certain variable (speed ratio, dressing depth, and cross-feed rate), and *V*_max⁡_ and *V*_min⁡_ are the maximum and minimum values of the independent variable. Additionally, ‘‘0.9” is its new maximum value (*new* max), and ‘‘0.1” is the variable's new minimum value (*new* min). The input pattern vectors are then formed, comprising 19 pairs of input/output ones for training the neural network on the basis of the previous mentioned experiments. The remaining 6 pairs are reserved for testing the trained network performance.

### 4.3. Training and Testing Performance Criterion

The training performance of the ANFIS model can be checked by the root mean square error (RMSE) as follows:

(12) $$\begin{array}{c} \text{RMSE}=\sqrt{\frac{1}{M}\sum_{z=1}^{M}{\left(S_{z}-Y_{z}\right)}^{2}}, \end{array}$$

where *M* is the total number of training patterns (19 patterns), *S*_*z*_ is the target value, and *Y*_*z*_ is the ANFIS output value.

Also, the testing performance can be checked by the error of network predictions. For the test data sets (6 patterns), neural network predictions are calculated. These are compared with the corresponding experimental values. The linear regression and statistical analysis is only effective for large quantities of data. In the current circumstances, it would have been better to use the root mean square error (RMSE) as presented in (12).

### 4.4. ANFIS Topology, Training, and Testing

Modeling of the process with an ANFIS model is composed of two stages: training and testing of the network with experimental data. The training data consist of dressing values for speed ratio, dressing depth and cross-feed rate, and corresponding specific energy. In all, 25 such data sets were used, of which, 19 data sets were selected randomly and used for training purposes, while the remaining 6 data sets (data marked by “∗” in Table 1) were presented to the trained network as new application data for verifying or testing the predictive accuracy of the network model. Thus, the network was evaluated using data that had not been used for training.

Since the model is based on a limited number of dressing conditions, it is necessary to ascertain whether the same model can predict the output parameter for the other conditions. Therefore, four overlapping data sets were prepared from the master set as shown in Table 1, and simulations were carried out individually for each of them.

The number of required rules and type of MF are very important considerations when solving actual problems using ANFIS network. To find the best network model that gives superior results in comparison with other networks topologies, a number of candidate networks with different number of rules and different MF types firstly were developed using the ANFIS editor of the Matlab 7.1 (14th release) software. Then, all ANFIS structures were trained based on the error goal (*RMSE*) of *0.001* and maximum number of *100* epochs. It means that the training epochs are continued until the RMSE fell below *0.001* or the epochs go up *100*. As the *RMSE* criterion for all networks is the same, their actions are comparable. Then their testing performances were compared, and the optimized model is selected based on its predictive accuracy in response to new input data in the testing phase when compared with experimental values.

**Figure 4:**
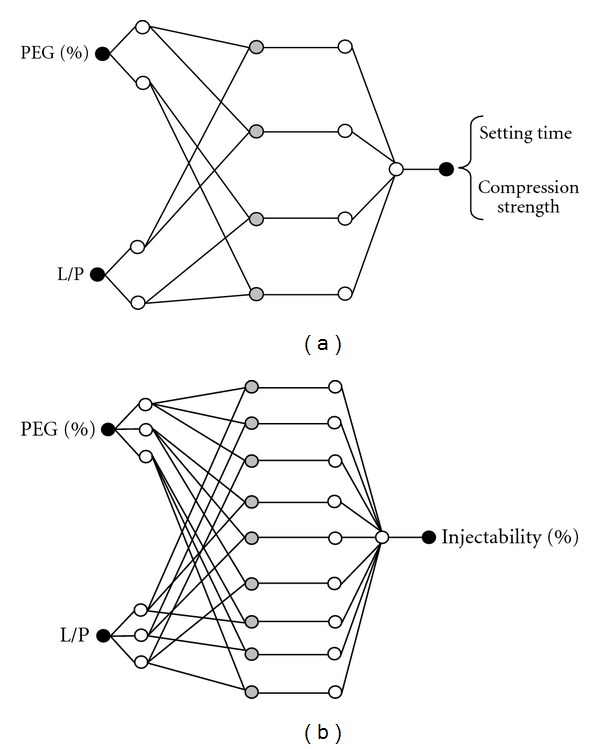
Structure of ANFIS models for (a) compression strength and setting time and (b) injectability.

By testing the various ANFIS structures with different number of membership functions, it obtained the optimal structure by trial and error method. The optimal structures have 8 membership functions for compression strength and setting time models as shown in Figure 4(a). Also, it has 18 membership functions for injectability model as shown in Figure 4(b).

**Figure 5:**
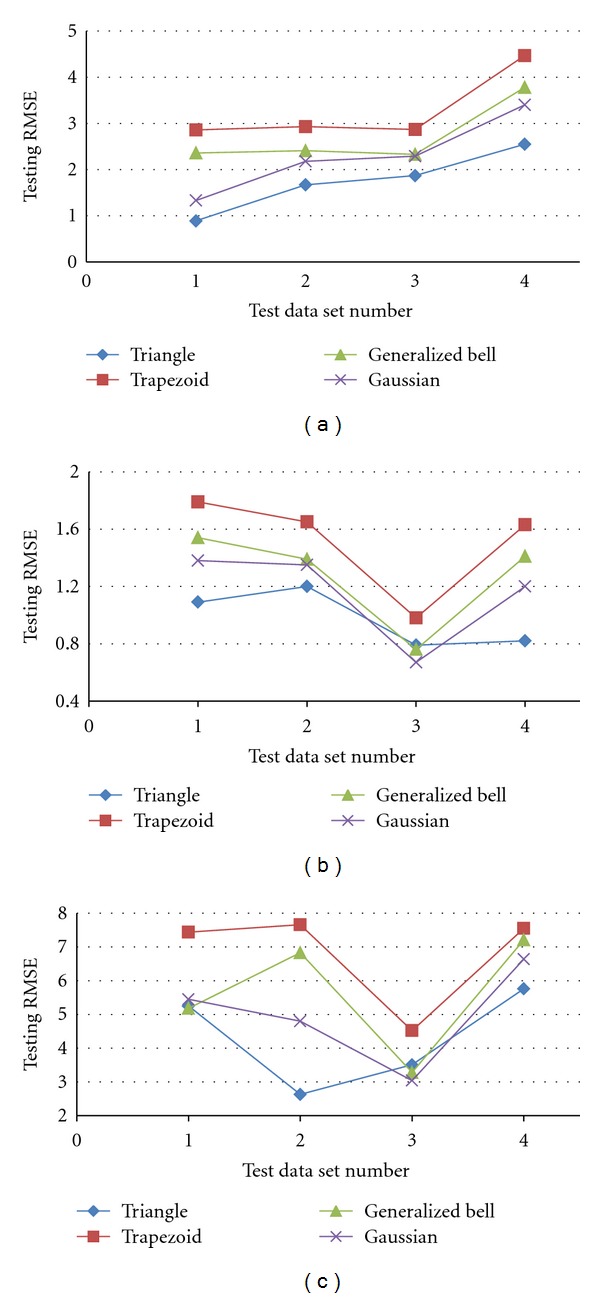
Testing RMSE of (a) setting time, (b) compression strength, and (c) injectability.

Also, different types of membership functions like bell, sigmoid, triangle, trapezoid, and Gaussian were tested.  Figure 5 shows the testing RMSE of three ANFIS models. 

Here four types of MF and four test data sets were assumed, and then the RMSE was presented in each case. Results show that the triangle function with constant fuzzy rules in comparison with others has minimum RMSE values for all models. Average errors are *0.97*, *1.74* and *4.29* for setting time, compression strength, and injectability models, respectively. Therefore, the triangle function with 8 rules is the best architecture for compression strength and setting time models, and also triangle function with 18 rules is the best architecture for injectability model.

**Figure 6:**
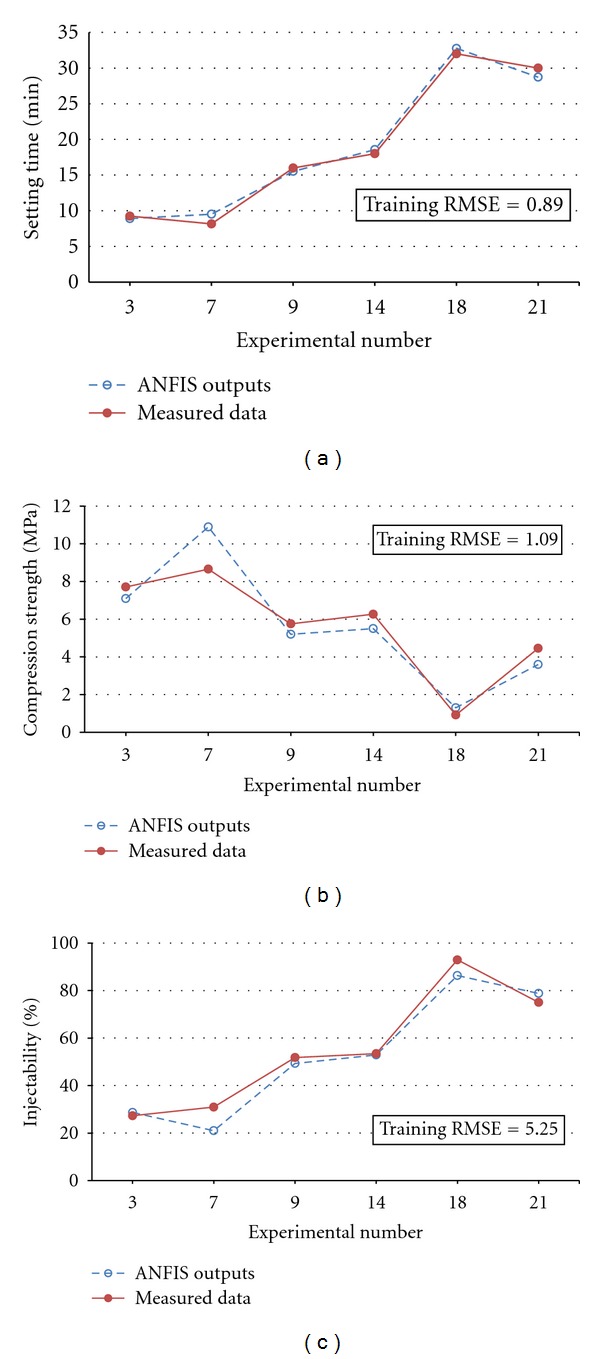
Comparison of the predicted values and measured data of (a) setting time, (b) compression strength, and (c) injectability for data test no. 1.

Comparison of the measured results and those of estimated values by ANFIS models for data test no. 1 and triangle MF are shown in Figure 6. It can be seen that the predicted results have a good agreement with experimental results for a wide range of data. They are acceptable, considering the limited amount of training data available and large error prone to measurements of calcium phosphate properties. Therefore, the adopted ANFIS can be used to acquire a function that maps input parameters to the desired process outputs.

## 5. Conclusions

In this study, an ANFIS model has been used to predict the mechanical properties, setting times, and injectability in CaPC. Considered input parameters were % PEG and liquid/powder ratio to determine the setting properties. The required training and validation data have been obtained from experimental observation. Three separate adaptive neurofuzzy inference systems with 8, 8, and 18 rules and triangle membership function were developed for compression strength, setting time, and injectability models, respectively. Novelty of this research is the use of neural network and fuzzy sets in modeling of synthesis of calcium phosphate bone cement which has not been worked as yet. ANFIS model provided an interesting method for modeling the setting properties. Despite the relatively small amount of data (25 conditions), the ANN model gave satisfying results. By adding more data, the ANN model can easily be expanded. If based on a larger amount of data, it might be possible to predict the output parameter with sufficient accuracy. The application of neural networks for the prediction of other setting properties might be of interest as well.

## Figures and Tables

**Table 1:** Experimental data of cements.

Exp. no.	Inputs		Outputs			Test data set			
	PEG (%)	*L/P* ratio (cc/gr)	Setting time (min)	Compression strength (MPa)	Injectability (%)	no. 1	no. 2	no. 3	no. 4
1	0	0.3	5.5	12.2	16.9		∗		
2	4	0.3	8	8.9	22			∗	
3	8	0.3	9.25	7.1	28.7	∗			
4	12	0.3	9.5	4.6	33.3		∗		
5	16	0.3	13	4.4	39.7				∗
6	20	0.3	15.75	3.1	46.5			∗	
7	0	0.35	8.15	10.9	21	∗			
8	4	0.35	12.5	6.3	39.1		∗		
9	8	0.35	16	5.2	49.3	∗			
10	12	0.35	18.75	4.9	61.6			∗	
11	16	0.35	22	3.6	75.2				∗
12	20	0.35	27.25	1.5	79.8			∗	
13	0	0.4	10	8.2	42.8				∗
14	4	0.4	18	5.5	52.9	∗			
15	8	0.4	21.25	4.8	66.3		∗		
16	12	0.4	25	3.2	75.7				∗
17	16	0.4	29.5	2.1	81.4			∗	
18	20	0.4	32	1.3	86.3	∗			
19	0	0.45	22.5	7.3	48.4				∗
20	4	0.45	27	5.6	67.9		∗		
21	8	0.45	30	3.6	78.8	∗			
22	12	0.45	33.5	2.8	80.3			∗	
23	16	0.45	35.25	1.3	90.5				∗
24	20	0.45	40.75	1.2	93.3		∗		
